# Genome-Wide Identification and Expression Analysis of Mitochondrial Dicarboxylate Carriers (DICs) in Medicago Under Aluminum Stress

**DOI:** 10.3390/plants14213250

**Published:** 2025-10-23

**Authors:** Chengcheng Yan, Xiaoqing Liu, Zhen Li, Yujie Lin, Zhenfei Guo, Yang Zhang

**Affiliations:** College of Grassland Science, Nanjing Agricultural University, Nanjing 210095, China

**Keywords:** aluminum stress, Al-responsive gene, DIC, Medicago, STOP1

## Abstract

Aluminum (Al) is solubilized as phytotoxic Al^3+^ in acidic soils, rapidly inhibiting root elongation. To detoxify Al, plant roots secrete organic acids that chelate the ion. The transcription factor SENSITIVE-TO-PROTON-RHIZOTOXICITY1 (STOP1) regulates the export, distribution and metabolism of organic acids, which is crucial for Al resistance. Plant DICARBOXYLATE-CARRIERs (DICs) located in the inner mitochondrial membrane are presumed to exchange the dicarboxylates. However, whether Al or STOP1 modulates *DIC* expression to coordinate the organic acid shuttle remains unclear. Here, in the model legume *Medicago truncatula*, we identified three *DIC* genes and twelve in tetraploid *Medicago sativa*. Phylogenetic analysis places all Medicago DICs in a clade with Arabidopsis AtDIC1 and AtDIC2, whereas *AtDIC3* lacks an ortholog in *M. truncatula*. Mining RNA-seq datasets followed by qRT-PCR validation showed that *MtDIC2* is upregulated by Al in roots in a MtSTOP1-dependent manner. Consistently, STOP1-binding motifs exist in the *MtDIC2* promoter, and MtSTOP1 binds to the *MtDIC2* promoter in yeast. Furthermore, *MsDIC2.4* shows an increase under Al treatment. Our study provides a genome-wide characterization of Medicago DICs and identifies *MtDIC2* as a candidate target of MtSTOP1, whose Al-responsive induction may enhance organic acid flux across the mitochondrial membrane.

## 1. Introduction

In addition to respiration and ATP generation, plant mitochondria also participate in photorespiration, supply carbon skeletons for anabolism, and modulate cellular redox balance [1,2]. Because these interconnected pathways span several cell compartments, hydrophilic metabolites must cross the inner mitochondrial membrane via members of the nuclear-encoded mitochondrial carrier family (MCF) [3,4,5]. Among them, DICARBOXYLATE-CARRIERs (DICs) mediate the transport of dicarboxylates [3,6,7]. In Arabidopsis, three of the six originally annotated UNCOUPLING-PROTEINs (AtUCP4–6) were renamed AtDIC1–3 after liposome assays revealed their capacity to transport malate, oxaloacetate, succinate, phosphate, thiosulfate, sulfate, and sulfite [7]. Subsequent analyses showed that AtDIC2 preferentially imports malate in exchange for citrate export [8]. The reduced growth of *Atdic2* mutants cannot be restored by compensatory mechanisms such as accelerated sucrose catabolism, increased citrate supply from fatty-acid β-oxidation, alternative carrier activities, or remodeling of the TCA cycle and amino acid pathways. Therefore, despite the existence of redundant transport routes such as DICARBOXYLATE/TRICARBOXYLATE-CARRIER (DTC) and SUCCINATE/FUMARATE-CARRIER (SFC) [5,9], AtDIC2 remains essential for maintaining energy, metabolic, and redox homeostasis.

Environmental stress rapidly disrupts mitochondrial metabolism in plants, underscoring the critical role of MCFs in stress tolerance [10,11]. During prolonged darkness, *AtDIC2* expression rises steadily; without it, compromised organic acid mobilization and NAD redox homeostasis accelerate leaf senescence [8]. Transcript profiling reveals that *AtDIC1* and *AtDIC2* are upregulated by several environmental stresses, including exposure to cold, salt, osmotic, drought, phosphate starvation, UV-B irradiation, and wounding [5]. Biotic stresses likewise trigger the coordinated upregulation of all three *AtDICs*, while submergence rapidly induces *AtDIC1*, *AtDIC2*, and their *Eucalyptus grandis* orthologs *EgDICs* [12]. This hypoxic induction is regulated by GROUP-VII-ETHYLENE-RESPONSE-FACTOR (ERFVIIs), whose oxygen-dependent turnover is controlled by the N-end rule pathway [12,13,14]. Accordingly, *AtDIC2* overexpression markedly enhances submergence tolerance, establishing its critical role in metabolic resilience under hypoxia [12].

Aluminum (Al) is the most abundant metal in the Earth’s crust. Under acidic conditions, it dissolves as toxic Al^3+^ ions, which rapidly inhibits root tips and reduces crop yields [15,16,17]. Within transient exposure, Al represses mitochondrial respiration, triggering ATP depletion, reactive oxygen species (ROS) formation, dissipation of the inner mitochondrial membrane potential, and an almost simultaneous arrest of root elongation [18,19]. Plants counter this toxicity by secreting organic acids—primarily malate and citrate—that chelate Al in the rhizosphere [20,21,22,23]. The Al-resistance transcription factor SENSITIVE-TO-PROTON-RHIZOTOXICITY1 (STOP1) transcriptionally induces *AL-ACTIVATED-MALATE-TRANSPORTER (ALMT)* and *MULTI-DRUG-AND-TOXINCOMPOUNDS-EXTRUSION (MATE)* expression to mediate malate and citrate secretion, respectively [24,25,26]. STOP1 also improves the ATP-binding cassette (ABC) transporter complex STAR1/ALS3 (STAR2) expression or protein abundance at the tonoplast to sequester Al into the vacuole, thereby restricting cytosolic toxicity [27,28,29,30,31]. In addition, STOP1 fine-tunes intracellular redox and metabolic balance [32,33]. SlSTOP1 confers Al tolerance by upregulating mitochondrial formate dehydrogenase *SlFDH*, which regenerates NAD^+^ from NADH [33]. In the *Atstop1* mutant, transcripts of the vacuolar malate importer *AtTDT*, the GABA-shunt enzymes *AtGDH1*, *AtGDH2* and *AtGAD1*, and the pH-stat enzymes *AtME1* and *AtME2* are all reduced [32]. Consistently, metabolite profiling reveals that sucrose accumulates, while succinate, pyruvate and GABA decline. AtSTOP1 binds to the *AtGDH1* and *AtGDH2* promoters, and mutation of these genes exacerbates Al sensitivity [34]. Thus, STOP1-mediated control of organic acid distribution and metabolism is important for Al tolerance. However, it remains unknown whether Al or STOP1 modulate *DIC* expression to coordinate organic acid exchange between mitochondria and cytosol.

Al-sensitive legume crops such as alfalfa (*Medicago sativa*, 2n = 4x = 32) and the model species barrel medic (*Medicago truncatula*, 2n = 2x = 16) suffer severe yield losses in acidic soils [35,36]. Recent work has begun to unravel the molecular mechanisms of their Al resistance. In *M. sativa*, Al-inducible genes including *MsMATE66*, *MsALS3*, *MsSTAR1*, and *MsGDH1* have been characterized [37]. Their *M. truncatula* orthologs are upregulated primarily through MtSTOP1 and its homolog MtSTOP4 [38]. Citrate efflux from Medicago roots is mediated by MtMATE66 [39], while the dehydrin MsDHN1 activates two aquaporins to promote oxalate secretion [40]. Additionally, the transcription factor MsMYB741 enhances flavonoid biosynthesis and exudation, further chelating external Al [41]. Furthermore, overexpression of MALATE-DEHYDROGENASE (MDH) or CITRATE-SYNTHASE (CS) markedly elevates Al resistance in *M. sativa* [42,43]. Hence, delineating the Medicago DIC family and determining their Al responsiveness might identify new targets for improving Al resistance by modulating mitochondrial-to-cytosolic organic acid flux.

In this study, we identified three *DIC* genes (*MtDIC1–3*) in *M. truncatula* and twelve in *M. sativa*. The phylogenetic tree, gene expansion patterns, sequence structures, and *cis*-regulatory elements of the DIC family were characterized. The response of DIC family genes to Al was further investigated using our previous RNA-seq datasets [37,38]. qRT-PCR revealed that MtSTOP1 drives Al-inducible *MtDIC2* expression in roots, while *MtDIC3* is transcriptionally repressed by Al but in a MtSTOP1-independent manner. Consistent with this, STOP1-binding motifs are present in the *MtDIC2* promoter. The *MsDIC2.4* expression also exhibits a significant increase under Al treatment. Collectively, our work establishes the Medicago DIC repertoire and pinpoints *MtDIC2* and *MsDIC2.4* as promising targets for enhancing adaptation of Medicago to acidic soils.

## 2. Results

### 2.1. Identification and Phylogenetic Analysis of the Dicarboxylate Carriers in Medicago

Using the three dicarboxylate carriers from Arabidopsis (AtDIC1–3) as query sequences, we searched for DIC homologs from 11 plant species. In the diploid *Medicago truncatula*, we identified three genes (*MtDIC1*–*3*), whereas the autotetraploid *Medicago sativa* contains 12 genes that were named based on their chromosomal locations (*MsDIC1.1*–*3.4*) (Figure S1 and Table S1). At the haploid genome level, *M. sativa* has the same number of DICs as *M. truncatula*. All Medicago DIC proteins range from 313 to 322 amino acids, exhibit isoelectric points of 9.85–9.96, and possess molecular weights of 33.4–34.5 kDa, which are highly similar to AtDIC1 and AtDIC2 (Table S2).

**Figure 1:**
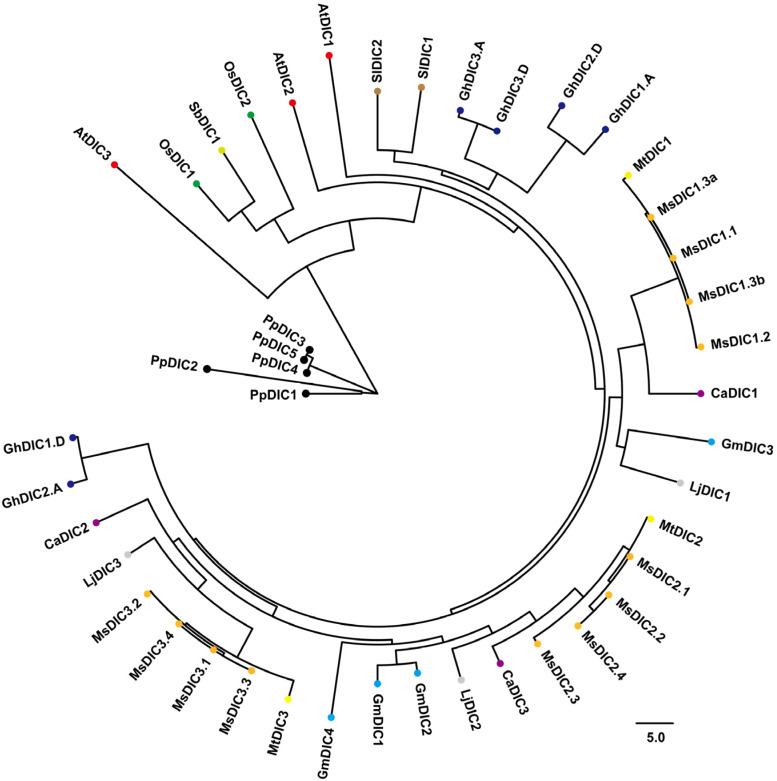
Phylogenetic tree of the DIC gene family. DIC protein sequences from *M. truncatula*, *M. sativa*, Arabidopsis, *L. japonicus*, soybean (*G. max*), chickpea (*C. arietinum*), tomato (*S. lycopersicum*), cotton (*G. hirsutum*), sorghum (*S. bicolor*), rice (*O. sativa*), and *P. patens* represented are marked by colored circles. The neighbor-joining tree was built with 1000 bootstrap replicates.

Subsequently, phylogenetic analysis was performed using DICs from Arabidopsis, *M. truncatula*, *M. sativa*, *Lotus japonicus*, soybean (*Glycine max*), chickpea (*Cicer arietinum*), tomato (*Solanum lycopersicum*), cotton (*Gossypium hirsutum*), sorghum (*Sorghum bicolor*), rice (*Oryza sativa*), and the moss *Physcomitrium patens* (Figure 1 and Figure S2, and Table S1). Consistent with their shared ancestry, each MtDIC grouped tightly with its four corresponding MsDIC members. MtDIC2 and MtDIC3 clustered more closely with each other than with MtDIC1. All MtDIC and MsDIC proteins clustered near AtDIC1 and AtDIC2 but were clearly separated from AtDIC3. AtDIC3, together with moss DICs, is phylogenetically distant from the other DICs. In addition, MtDICs and MsDICs clustered closely around DICs from other legumes, such as *L*. *japonicus*, soybean, and chickpea, indicating a shared evolutionary history and conserved molecular function.

### 2.2. Synteny Analysis of DICs in the Genome of Medicago

**Figure 2:**
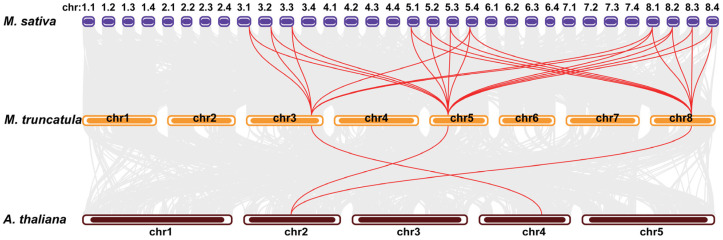
Syntenic analysis of *DIC* genes among *M. sativa*, *M. trucatula*, and Arabidopsis. Red lines connect orthologous *DIC* pairs. Grey lines connect other orthologous pairs.

To further examine the evolutionary relationships of the *DICs*, we performed inter-species synteny analyses among *M. truncatula*, *M. sativa*, and Arabidopsis. Three orthologous pairs were identified between *M. truncatula* and Arabidopsis, while 25 orthologous pairs were detected between *M. truncatula* and *M. sativa* (Figure 2 and Table S3). Consistent with the phylogenetic results (Figure 1), no *MtDIC* was found orthologous to *AtDIC3* (Figure 2 and Table S3). We next examined the synteny within each species. Three paralogous pairs were observed among the *MtDICs* within *M. truncatula* (Figure S3a and Table S4). In *M. sativa*, 17 paralogous pairs were discovered (Figure S3b). Notably, none of the *MsDIC1* members showed paralogous to *MsDIC2* or *MsDIC3* members.

### 2.3. Protein Motifs, Gene Structures and Cis-Elements in DIC Promoters

**Figure 3:**
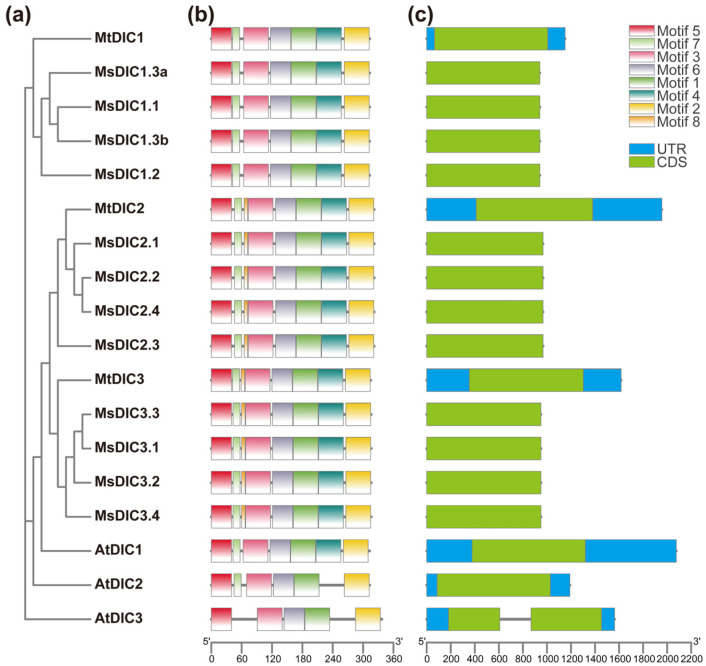
Protein motifs and gene structures of the DIC family in Medicago and Arabidopsis: (**a**) neighbor-joining tree (1000 bootstraps); (**b**) distribution of conserved motifs; (**c**) gene structures.

Next, we aligned these DIC protein sequences from *M. truncatula*, *M. sativa*, and Arabidopsis with human UCP1 (HsUCP1) of known cryo-electron microscopy structure [44] (Figure S4). It showed that MtDICs and MsDICs are more closely related to AtDIC1/AtDIC2 than to AtDIC3. All DICs contain three tandem repeats, each comprising two hydrophobic transmembrane α-helices linked by a long hydrophilic matrix loop [4,45,46]. Within the nine predicted α-helices (Hs), transmembrane segments H1, H4, and H7 carry the conserved PX[DE]XX[RK] motif, whose charged residues form the matrix salt bridge network. Adjacent to the transmembrane segments H3, H6, and H9, there are the conserved [YF][DE]XX[KR] motif that can form another salt bridge called the cytoplasmic salt bridge network. Furthermore, MEME motif discovery identified eight conserved motifs in all these DICs (Figure 3a,b and Figure S5). Motifs 5, 3, 6, 1, and 2 are shared by all Medicago and Arabidopsis DICs. Motifs 7 and 4 are additionally present in AtDIC1 and all Medicago DICs, whereas Motif 8 is restricted to MtDIC2/MsDIC2s and MtDIC3/MsDIC3s.

Gene structure analysis revealed that all Medicago *DIC* genes, like *AtDIC1* and *AtDIC2*, comprise a single exon (Figure 3c). *AtDIC3* uniquely contains one intron and two exons. *MsDIC* genes displayed no annotated 5′- and 3′-UTRs, which may be due to incomplete gene annotation rather than biological loss.

**Figure 4:**
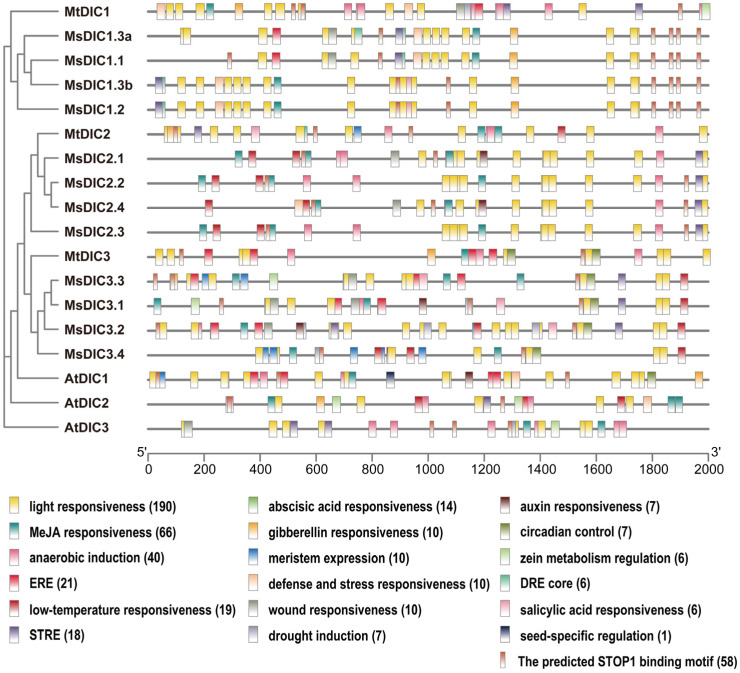
*Cis*-element landscape of *DIC* promoters in Medicago and Arabidopsis. PlantCare database was used to scan the 2 kb upstream regions of Medicago and Arabidopsis *DIC* genes.

To explore the transcriptional regulatory mechanisms, we scanned the 2 kb promoter regions of Medicago and Arabidopsis *DIC* genes using the PlantCARE database (Figure 4 and Figure S6). Light-responsive elements (3-AF1 binding site, AE-box, Box 4, chs-CMA1a/2a, G-box, GT1-motif, I-box, MRE, Sp1, TCCC-motif, and TCT-motif) are the most abundant class, with Box 4 present in every Medicago *DIC* promoter. Hormone-responsive motifs for MeJA, ethylene, ABA, gibberellin, auxin and salicylic acid, together with stress- and development-associated elements, are also widespread. The TGACG-motif, MeJA-responsiveness element, is present in all DIC genes of Medicago and Arabidopsis. Significantly, the Al-resistance transcription factor STOP1 binding motif, identified from AtSTOP1 DAP-seq data [47], is found in the promoters of all Medicago and Arabidopsis *DIC* genes (Figure 4 and Table S5), suggesting the DIC family in Al signaling.

### 2.4. Expression Analysis of DIC Genes in Medicago Under Al Treatment

**Figure 5:**
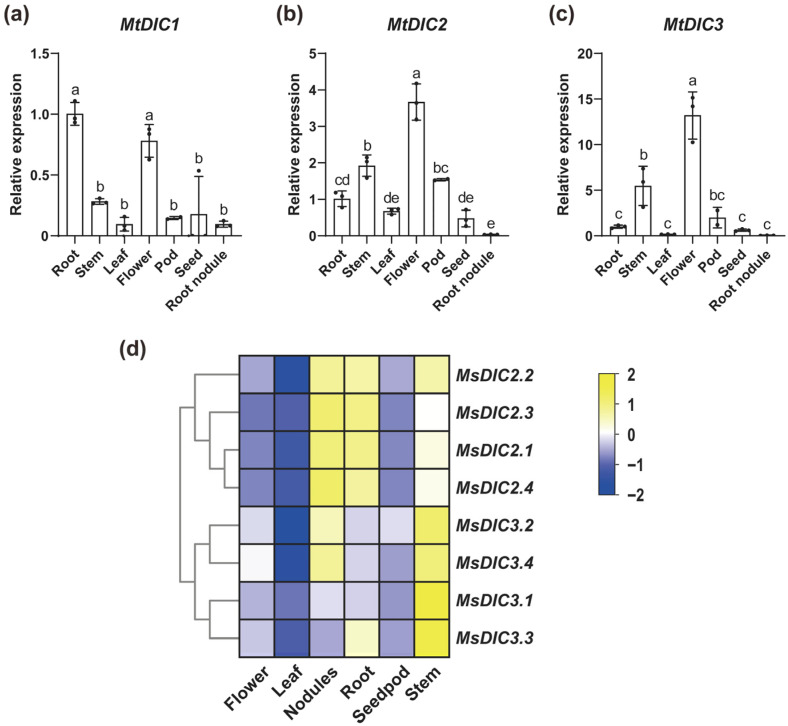
Tissue-specific expression of Medicago *DIC* genes: (**a**–**c**) qRT-PCR quantification of *MtDIC1*, *MtDIC2* and *MtDIC3* in R108 (WT) tissues (n = 3 independent pools). Expression is normalized to roots. Means ± SD. Different letters indicate significant differences (one-way ANOVA, Tukey’s test, *p* < 0.05). (**d**) Z-score heatmap of *M. sativa DIC* expression across tissues.

We performed qRT-PCR analysis to determine the tissue-specific expression of *DICs* in *M. truncatula*. *MtDIC1* transcripts accumulated predominantly in roots and flowers, while *MtDIC2* and *MtDIC3* were most highly expressed in flowers (Figure 5a–c). In roots, *MtDIC2* transcripts were the most abundant (mean 2^−ΔCt^ = 0.0691), followed by *MtDIC3* (0.0042), whereas *MtDIC1* showed the lowest (0.0014). Analyzing public RNA-seq data from *M. sativa* [48] further revealed that *MsDIC2* members are highly expressed in roots, nodules and stems, with *MsDIC2.4* showing the highest FPKM, while *MsDIC3* transcripts are most abundant in stems (Figure 5d). Moreover, transcripts of *MsDIC1s* were too low to be detected, indicating that *MtDIC1* and *MsDIC1s* are the least expressed in these tissues.

**Figure 6:**
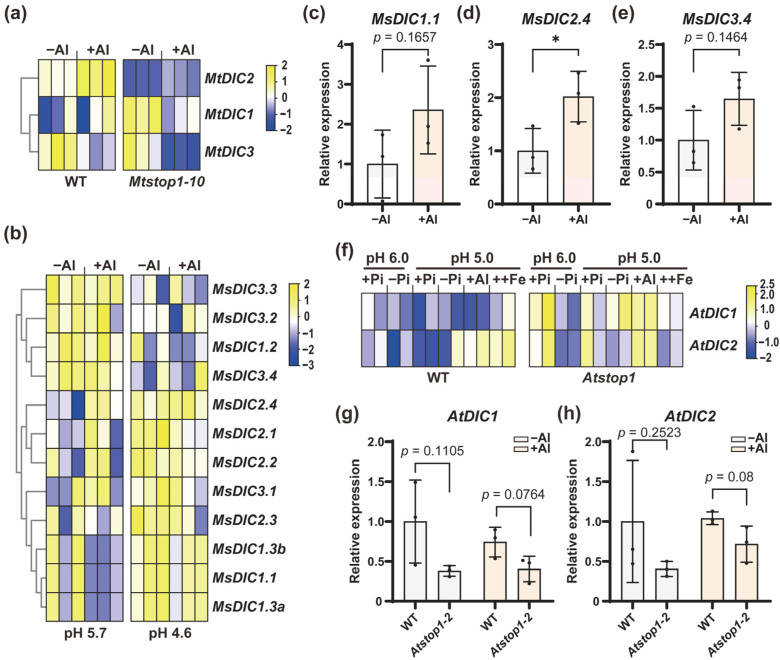
Al-responsive expression profiles of Medicago and Arabidopsis *DIC* genes. Heatmaps depicting the normalized gene expression (z-score) of *MtDICs* (**a**), *MsDICs* (**b**), and *AtDICs* (**f**) under Al stress. qRT-PCR analysis of *MsDIC1.1* (**c**), *MsDIC2.4* (**d**), and *MsDIC3.4* (**e**) in root tips of WT (XinJiangDaYe), as well as *AtDIC1* (**g**) and *AtDIC2* (**h**) in total roots of WT (Col-0) and *Atstop1-2* (n = 3 independent pools). Seven-day-old seedlings treated ± 5 µM AlCl_3_ in 0.5 mM CaCl_2_ (pH 5.0, 6 h). Expression is relative to WT under 0 µM AlCl_3_ conditions. Means ± SD; * *p* < 0.05 (two-tailed *t*-test); ns, not significant.

To further investigate whether Medicago *DICs* respond to Al, we analyzed our previous RNA-seq datasets in *M. truncatula* and *M. sativa* [37,38]. In WT (R108), we observed that *MtDIC2* was significantly upregulated by Al among the differentially expressed genes (DEGs) (fold change, FC = 2.39; false discovery rate, FDR < 0.05) (Figure 6a). In contrast, *MtDIC3* was slightly downregulated by Al (FC = 0.60, FDR = 0.136, *p* = 0.034) but was not identified as a DEG. In a previous stress time-course [49], *MtDIC2* expression was also induced by cold and drought, while *MtDIC3* responded to salt (Figure S7). In *M. sativa*, *MsDIC2.4* showed a modest induction under Al at pH 5.7 (FC = 1.37, FDR = 0.718, *p* = 0.012) relative to pH 5.7 without Al. However, this induction did not reach the threshold for DEGs. Both *MsDIC2.4* and *MsDIC2.1* were slightly induced by low pH alone (pH 4.6 vs. pH 5.7), with FC of 1.39 and 1.52, FDR of 0.482 and 0.419, and *p* values of 0.004 and 0.003, respectively (Figure 6b). qRT-PCR analysis confirmed that *MsDIC2.4* expression can be significantly induced by Al, whereas the expression of *MsDIC1.1* and *MsDIC3.4* was not (Figure 6c–e). Then, we analyzed RNA-seq data in Arabidopsis [50]. *AtDIC2* was slightly induced by Al and excess Fe at pH 5 (with +Pi pH 6 as control), although it did not reach the threshold for DEGs (Figure 6f). This induction was attenuated in the *Atstop1* mutant. qRT-PCR analysis revealed that both *AtDIC1* and *AtDIC2* exhibited slightly reduced expression levels in the *Atstop1-2* compared to WT (Col-0) under Al treatment (Figure 6g,h). Furthermore, in previous DAP-seq data [47], we found that the upstream and downstream regions of *AtDIC2* were enriched with AtSTOP1, which contains the predicted AtSTOP1 binding motif (Figure S8), indicating that AtSTOP1 directly targets *AtDIC2* to regulate its expression.

### 2.5. MtSTOP1 Upregulates MtDIC2 Expression in Roots Under Al Stress

**Figure 7:**
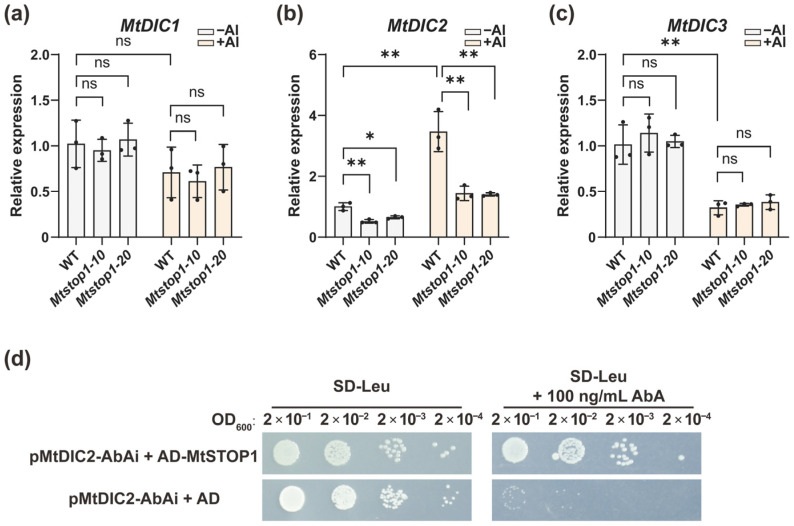
MtSTOP1 is required for Al-induced *MtDIC2* expression in roots. qRT-PCR of *MtDICs* in root tips (**a**–**c**) of WT (R108), *Mtstop1-10*, and *Mtstop1-20* (n = 3 independent pools): (**a**–**c**) Four-day-old seedlings treated ± 5 µM AlCl_3_ in 0.5 mM CaCl_2_ (pH 5.0, 6 h). Expression is relative to WT under 0 µM AlCl_3_ conditions. Means ± SD; * *p* < 0.05, ** *p* < 0.01 (two-tailed *t*-test); ns, not significant. (**d**) Interaction between MtSTOP1 and the *MtDIC2* promoter in the yeast one-hybrid assay. Y1H Gold yeast strains carrying the pMtDIC2-AbAi were transformed with either AD-MtSTOP1 or the empty vector (AD alone). These transformed yeast strains were then subjected to a 10-fold serial dilution and cultured on SD-Leu medium or SD-Leu medium supplemented with Aureobasidin A (AbA). Growth on SD-Leu medium plus AbA indicates a positive interaction.

The RNA-seq data showed that among DEGs between WT (R108) and the knockout mutant *Mtstop1-10*, the expression of *MtDIC2* was significantly suppressed in the knockout mutant *Mtstop1-10* under both control (FC = 0.45, FDR < 0.05) and Al-treated (FC = 0.22, FDR < 0.05) conditions (Figure 6a). qRT-PCR analysis using root tip confirmed that Al-inducible *MtDIC2* expression in WT, whereas induction was abolished in two independent knockout lines, *Mtstop1-10* and *Mtstop1-20* (Figure 7a–c). Conversely, Al repressed *MtDIC3*, yet mutation of *MtSTOP1* unaffected its expression (Figure 7c). Interestingly, MtSTOP1-regulated expression of *MtDIC2* was absent in leaves (Figure S9), and *MtDIC1* transcripts were below detection in seedling leaves. Subsequently, we performed a yeast one-hybrid assay to determine whether MtSTOP1 could bind to the *MtDIC2* promoter. The yeast strain, whose genome had previously been integrated with the linearized pMtDIC2-AbAi, was then transformed with either an empty vector (AD alone) or AD- MtSTOP1. All transformed yeast strains grew well on SD-Leu medium (Figure 7d). However, only the yeast transformed with the AD-MtSTOP1 fusion grew robustly on SD-Leu medium supplemented with Aureobasidin A (AbA). This result indicates that MtSTOP1 can activate the expression of the reporter gene AbA^r^, suggesting that MtSTOP1 can bind to the *MtDIC2* promoter in yeast.

Since plant DICs share high homology with UCPs [4,51], we also examined *MtUCP* expression under Al stress, yet none of them responded appreciably (Figure S10). In addition, DTC and SFC are single-copy genes in Arabidopsis and *M. truncatula* that mediate mitochondrial organic acid transport [9]. Exposure to Al caused a modest induction of *MtDTC* expression (FC = 1.79, FDR = 0.073, *p* = 0.013) in a MtSTOP1-independent manner, whereas *MtSFC1* expression was unaffected.

## 3. Discussion

In Arabidopsis, 58 members of MCF have been identified [6,9,45], among which the three DIC proteins (AtDIC1–3) facilitate dicarboxylate transport [4,7,8]. AtDIC1 and AtDIC2 are 70% identical, while AtDIC3 is only 55–60% identical to AtDIC1 and AtDIC2 [7]. A mitochondrial proteomic survey revealed that *AtDIC3* is expressed at significantly lower levels than *AtDIC1* and *AtDIC2* [4], and root RNA-seq likewise failed to detect *AtDIC3* transcripts (Figure 6f). We identified three *MtDIC* genes in *M. trucatula* and twelve *MsDIC* genes in *M. sativa*. All legume DICs form a single clade near to AtDIC1 and AtDIC2 but clearly distinct from AtDIC3 (Figure 1 and Figure S2). The Medicago DIC proteins are 313–322 residues long, closely matching AtDIC1 and AtDIC2 (both 313 aa) and markedly shorter than AtDIC3 (337 aa) (Table S2). Synteny analysis revealed no ortholog of *AtDIC3* in *M. trucatula* (Figure 2 and Table S3). Furthermore, every Medicago DIC, like *AtDIC1* and *AtDIC2*, is encoded by a single exon, whereas *AtDIC3* uniquely contains two exons separated by one intron (Figure 3c). Collectively, these data suggest that Medicago DICs are functionally aligned with AtDIC1 and AtDIC2 rather than with the divergent AtDIC3. Functional assays showed that AtDIC1 and AtDIC2 transport dicarboxylates, phosphate and arsenate far more efficiently than AtDIC3 does [7]. Moreover, both in isolated mitochondria and in proteoliposome studies further revealed that AtDIC2 operates as a malate-importer/citrate-exporter antiporter [8]. However, whether the Medicago DIC exhibit the same substrate preferences and transport directionality remains to be determined.

All MCFs share an architecture of three tandem repeats, each repeat comprising two hydrophobic transmembrane helices linked by a characteristic motif, PX[D/E]XX[K/R]X[K/R] (20–30 residues) [D/E]GXXXX[W/Y/F][K/R]G [3,46,52,53]. Consistent with this, MtDIC and MsDIC sequences fold into nine predicted α-helices that contain the three repeated motifs (Figure S4). Strikingly, MEME Motif 8 appears exclusively in MtDIC2/MsDIC2s and MtDIC3/MsDIC3s (Figure 3a,b), and phylogenetic analysis places these sequences in a single clade separate from MtDIC1/MsDIC1s (Figure 1 and Figure S2). In addition, no *MsDIC1* member is paralogous to any *MsDIC2* or *MsDIC3* member (Figure S3b). These suggest that MtDIC2/MsDIC2s and MtDIC3/MsDIC3s constitute a subgroup that has diverged independently from the MtDIC1/MsDIC1s. Expression profiling mirrors this divergence. *MtDIC2* and *MtDIC3* transcripts accumulate to markedly higher levels than *MtDIC1*, and are both most abundant in flowers (Figure 5a–c); moreover, transcripts of *MsDIC1* in tissues and *MtDIC1* in seedling leaves are virtually undetectable (Figure 5d and Figure S9). Three paralogous pairs were observed among the *MtDICs* (Figure S3a and Table S4), while 17 paralogous pairs were discovered in *M. sativa*, confirming the duplication of *DIC* genes in Medicago, which is likely caused by whole-genome duplication (WGD) [54].

Al toxicity is tightly linked to mitochondrial dysfunction [19,55]. Plants detoxify the rhizosphere Al by secreting organic acids, a process that consumes carbon and energy [20,23]. As MCF transporters control metabolite and energy homeostasis, they are likely pivotal to Al resistance [11,18,56]. Among them, DIC, DTC, and SFC proteins are candidates for shuttling organic acids within mitochondrial metabolism [8,9]. In this study, we observed that *MtDIC2* expression is significantly up-regulated within 6 h of Al treatment (Figure 6a and Figure 7b), *MtDTC* shows a modest Al induction, and *MtSFC1* remains unchanged (Figure S10). Likewise, *MsDIC2.4* expression exhibits an increase under Al (Figure 6b,d). AtDIC1–3, originally designated AtUCP4–6, share high homology with AtUCP1–3 [4,51]. Previous work has shown that AtUCP1 localizes to mitochondria and displays uncoupling activity [10,57]. AtUCP1 and AtUCP2 also transport aspartate, glutamate, and other dicarboxylates [51]. However, profiling the three *MtUCP* genes under Al stress revealed no significant response (Figure S10), implying they do not participate in Al-triggered shuttling of organic acids in mitochondria.

AtSTOP1 confers Al resistance by transcriptionally regulating *AtALMT1*, *AtMATE*, and *AtSAUR55* for organic acid secretion [24,26,58] and by inducing *AtALS3*, *AtGDH1*, and *AtGDH2* for internal detoxification [34]. Additionally, STOP1 adjusts intracellular redox, metabolite, and pH balance by regulating FDH, TDT, GABA-shunt enzymes, and the pH-stat enzymes [32,33]. In *M. truncatula*, MtSTOP1 primarily modulates Al resistance by inducing *MtMATE66*, *MtALS3*, *MtSTAR1*, and *MtGDH1* expression [38]. Here, we found that *AtDIC2* expression is slightly reduced in *Atstop1* under Al treatment (Figure 6f,h), while the induction of *MtDIC2* expression by Al is significantly abolished in *Mtstop1* mutants (Figure 6a and Figure 7b). Supporting these observations, AtSTOP1 occupancy is enriched across both upstream and downstream regions of *AtDIC2*, as identified by AtSTOP1 DAP-seq [47]. Each contains the canonical STOP1-binding motif (Figure S8), suggesting that *AtDIC2* is transcriptionally controlled by AtSTOP1. The STOP1-binding motif is also present in the *MtDIC2* promoter (Figure 4 and Table S5). Additionally, MtSTOP1 has been shown to bind to the *MtDIC2* promoter in yeast (Figure 7d). These findings collectively suggest that *MtDIC2* is a candidate target of MtSTOP1.

STOP1 accumulation under Al stress is tightly associated with downstream gene expression [59]. On the one hand, AtSTOP1 stability is negatively regulated by ubiquitin-mediated proteolysis through REGULATIONOF-ATALMT1-EXPRESSION1 (AtRAE1) and its homolog [60,61], while the oxidative modification accelerates this degradation [55]. On the other hand, the THO/TREX complex positively regulates AtSTOP1 abundance [62,63], and phosphorylation further promotes its accumulation [64,65,66]. Additionally, SUMOylation fine-tunes AtSTOP1 accumulation and transcriptional activity [67]. In *M. truncatula*, although *MtSTOP1* transcripts show a slight increase in the very apex of roots, the protein might also be modulated by Al at the post-translational level through MtRAE1-mediated degradation [38]. In this study, we observed that MtSTOP1-regulated expression of *MtDIC2* is completely absent in leaves (Figure S9). This likely reflects insufficient MtSTOP1 levels in leaves to activate *MtDIC2* expression. Low pH also triggers STOP1 accumulation [68]. In *M. sativa*, the expression of MtSTOP1-regulated orthologous genes is induced by Al, and their expression is also responsive to acidic pH [37,38]. Here, we found that *MsDIC2.4* shows an increase under Al at both pH 5.0 and pH 5.7, despite the different Al speciation present at these pH levels (Figure 6b,d and Figure S11), while both *MsDIC2.4* and *MsDIC2.1* are induced by low pH.

In addition to Al and low pH, AtSTOP1 accumulates under Fe excess caused by Pi deficiency [50,69]. Here, we found a modest induction of *AtDIC2* under excess Fe (Figure 6f). *AtDICs* are transcriptionally activated by a range of abiotic and biotic stresses [5,10]. Similarly, the expression of *MtDIC2* is rapidly upregulated by cold and drought within 2–6 h, followed by a decrease at 12 h, whereas *MtDIC3* is induced by salt only after 12 h of treatment (Figure S7). Submergence rapidly induces *AtDIC1* and *AtDIC2* expression via the hypoxia-responsive ERFVIIs [12]. Hypoxia induces *STOP1* expression, and cellular redox perturbations influence STOP1 protein levels [55,70]. Additionally, STOP1 itself is implicated in tolerance to salt, drought, hypoxia, and a spectrum of other stresses [70,71,72]. Thus, *DICs* might be the targets of STOP1 under these diverse stresses. Al, Fe, heavy metals, and other stresses often cause oxidative damage [55,73,74]. The proposed function of DICs is associated with the transport of organic acids into or out of the mitochondria, thereby maintaining redox homeostasis [8]. Nevertheless, *cis*-element profiling reveals light-, hormone-, stress-, and development-associated motifs across all Medicago and Arabidopsis DIC promoters (Figure 4), indicating that additional transcription factors might also contribute to their regulation.

In summary, our research has characterized the Medicago DIC gene family and identified *MtDIC2* and *MsDIC2.4* as potential targets for improving Medicago’s tolerance to acidic soils. Further investigation is required to explore the effect of increasing its expression.

## 4. Materials and Methods

### 4.1. Identification of DIC Gene Family in Medicago

Protein sequences of AtDIC1, 2, and 3 were obtained from the Arabidopsis Araport11 database and used as BLASTP queries against *Medicago truncatula* A17 r5.0 genome [75], the *Medicago sativa* cv. XinJiangDaYe genome [76] or against eight additional plant genomes (*Lotus japonicus* Lj1.0v1, *Glycine max* Wm82.a4.v1, *Cicer arietinum* v1.0, *Solanum lycopersicum* ITAG2.4, *Gossypium hirsutum* v2.1, *Sorghum bicolor* v3.1.1, *Oryza sativa* v7.0, and *Physcomitrium patens* v3.3). The chromosomal locations of Medicago DICs were extracted from the corresponding assemblies and plotted with the TBtools-II software [77].

### 4.2. Phylogenetic and Synteny Analysis

DIC proteins were aligned using the MUSCLE method (default settings). The trimmed alignment was used to construct a neighbor-joining (NL) or maximum likelihood (ML) phylogenetic tree (1000 bootstrap replicates) in MEGA X [78]. MCScanX was used to infer gene synteny in TBtools-II.

### 4.3. Protein Motif Prediction and Gene Structure Survey

Physicochemical properties (molecular weight and isoelectric point) of Medicago and Arabidopsis DICs were calculated with ExPASy ProtParam (https://web.expasy.org/protparam/ (accessed on 21 October 2025)). They were aligned to human HsUCP1 and visualized with ESPript 3.0 (https://espript.ibcp.fr/ESPript/ESPript/index.php (accessed on 21 October 2025)). MEME v5.5.8 (http://meme-suite.org/ (accessed on 2 July 2025)) was used to predict the conserved motifs (maximum motifs = 8; otherwise default). The gene structures were extracted from the *M. truncatula* A17 r5.0, *M. sativa* XinJiangDaYe, and Arabidopsis Araport11 annotations. The results of protein motif and gene structure analysis were plotted using TBtools-II.

### 4.4. Cis-Element Profiling

Two-kb upstream regions (from the start codon) of Medicago and Arabidopsis DICs were scanned for *cis*-regulatory elements using the PlantCARE database (https://bioinformatics.psb.ugent.be/webtools/plantcare/html/ (accessed on 21 October 2025)). Results were visualized with TBtools-II.

### 4.5. Plant Materials and Growth Conditions

*M*. *truncatula* ecotype R108, *M*. *sativa* cultivar XinJiangDaYe, and Arabidopsis ecotype Col-0 were employed as the wild-type (WT) controls in this study. The *Mtstop1-10* and *Mtstop1-20* mutants were previously generated using the CRISPR/Cas9 system [38]. The T-DNA insertion line SALK_114108 (*Atstop1-2*) was procured from the Arabidopsis Biological Resource Center. Seedlings were grown in a growth chamber set at 22 °C, with a light cycle of 14 h of illumination and 10 h of darkness.

### 4.6. RNA-Seq Data Analysis

Our previous RNA-Seq data were analyzed to study gene expression under Al stress in *M. truncatula* [38] and *M. sativa* [37]. Differential expression analysis was performed using DESeq2 with a significance threshold of false discovery rate (FDR) < 0.05 and a fold change (FC) > 2 or <0.5. Additional transcriptome datasets were downloaded from NCBI GEO to examine expression profiles in different *M. sativa* tissues [48], Arabidopsis Al responses [50], and *M. truncatula* stress responses [49]. Expression values were calculated as log_2_ (FPKM/TPM + 1) and heatmaps were generated using the z-score normalization with TBtools-II.

### 4.7. Real-Time Quantitative Reverse-Transcription PCR (qRT-PCR) Analysis

To profile the tissue-specific expression of *MtDICs*, total RNA was extracted from *M. truncatula* R108 tissues, including roots of 5-day-old seedlings, stems and leaves of 2-week-old seedlings, and flowers, pods, seeds, and root nodules of 12-week-old plants, using a plant RNA isolation kit (Vazyme, Nanjing, China). For Al-stress assays, Medicago species were cultivated in a 0.5 mM CaCl_2_ solution before Al treatment, while Arabidopsis were grown on 1/2 MS medium with 1.2% agar and 1% sucrose at pH 5.7. Subsequently, 4-day-old WT (R108), *Mtstop1-10*, and *Mtstop1-20* seedlings, as well as 7-day-old XinJiangDaYe, WT (Col), and *Atstop1-2* mutants were pretreated with 0.5 mM CaCl_2_ at pH 5.0 for 6 h and then exposed to 0 or 5 μM AlCl_3_ in the same solution for 6 h. RNA was also extracted from root segments (0–1 cm) for Medicago species and from total roots for Arabidopsis. To determine MtSTOP1-dependent regulation in shoots, leaves of 2-week-old WT (R108) and *Mtstop1-10* were collected. RNA was reverse-transcribed with a 1st strand cDNA synthesis kit (Vazyme). qRT-PCR was performed using the SYBR Green reagent (Vazyme). *MtGAPDH* (MtrunA17Chr4g0057861) [38] was identified as the optimal internal reference gene for *M*. *truncatula* using the Normfinder (Table S6). *MsGAPDH* (MS.gene033342) and *AtUBQ10* (AT4G05320) [60] were used as internal reference genes for *M*. *sativa* and Arabidopsis, respectively. Primers are listed in Table S7. Relative expression levels were calculated using the 2^−ΔΔCt^ method.

### 4.8. Yeast One-Hybrid Assay

The promoter of *MtDIC2* was cloned into the pAbAi vector. The coding sequence (CDS) of MtSTOP1 was in-frame fused with the GAL4 activation domain of the yeast expression vector pGADT7. The primers used for these constructions are listed in Table S7. The yeast one-hybrid assay was performed according to the manufacturer’s instructions (Coolaber, Beijing, China). The transformed yeast cells were plated onto synthetic defined (SD) medium lacking leucine (Leu) and supplemented with either 0 or 100 ng/mL Aureobasidin A (AbA) to test the interaction for 3 days.

### 4.9. Aluminum Speciation Analysis

The speciation of Al was simulated using MINEQL+ 5.0 with default settings. Simulations were conducted for Al treatments at pH levels of 4.6, 5.0, and 5.7. The model input included the complete chemical composition of the solution, which contained 5 μM AlCl_3_ and 0.5 mM CaCl_2_.

### 4.10. Statistical Analysis

qRT-PCR was performed on three independent plant pools. Values are means ± SD. Statistical significance was determined by one-way ANOVA followed by Tukey’s test for more than two groups or a two-tailed *t*-test for two groups.

## Data Availability

Data are contained within the article and Supplementary Materials.

## Supplementary Materials

The following supporting information can be downloaded at: https://www.mdpi.com/article/10.3390/plants14213250/s1, Figure S1: Chromosomal locations of *MtDIC* and *MsDIC* genes; Figure S2: The maximum likelihood phylogenetic tree of the DIC gene family; Figure S3: Syntenic analysis of the DIC gene family in the genomes of *M. truncatula* and *M. sativa*, respectively; Figure S4: Multiple sequence alignment of DIC proteins; Figure S5: Sequence logos of the eight conserved motifs identified in Medicago and Arabidopsis DIC proteins; Figure S6: Heatmap showing the quantity of *cis*-acting elements in Medicago and Arabidopsis *DIC* promoters; Figure S7: Heatmap of *MtDIC* expression under cold, drought, and salt stresses; Figure S8: AtSTOP1-binding peaks detected by DAP-seq in upstream and downstream regions of *AtDIC2*; Figure S9: The expression of *MtDIC2* and *MtDIC3* in *Mtstop1* leaves remained unchanged relative to WT; Figure S10: Heatmap of *MtUCP*, *MtDTC*, and *MtSFC1* expression under Al stress in WT and *Mtstop1-10*; Figure S11: Effects of pH on Al speciation; Table S1: The DIC gene family members used for phylogenetic tree construction; Table S2: The protein lengths, molecular weights, and isoelectric points of the DIC gene family in *M. truncatula*, *M. sativa* and Arabidopsis; Table S3: The ortholog pairs of DIC family genes in the genomes of *M. trucatula*, *M. sativa*, and Arabidopsis; Table S4: The paralog pairs of DIC family genes in the genomes of *M. trucatula* and *M. sativa*; Table S5: The predicted STOP1 binding motifs in the promoters of DIC gene family members in *M. trucatula*, *M. sativa*, and Arabidopsis; Table S6: Identification of the optimal internal reference gene by Normfinder; Table S7: Primers used in this study.

## Abbreviations

The following abbreviations are used in this manuscript:

chr	Chromosome
RNA-seq	RNA sequencing
qRT-PCR	Real-time quantitative reverse transcription PCR
FC	Fold change
FDR	False discovery rate
DAP-seq	DNA affinity purification sequencing
