# Supplementary material for: Genome-Wide Identification and Expression Analysis of Mitochondrial Dicarboxylate Carriers (DICs) in Medicago Under Aluminum Stress
Source: Plants (Basel). 2025 Oct 23;14(21):3250. doi: 10.3390/plants14213250 (PMC12608790; doi:10.3390/plants14213250)
Supplement: Supplementary file 1 [file plants-14-03250-s001.zip › Figure S1-S11.pdf]

Figure S1

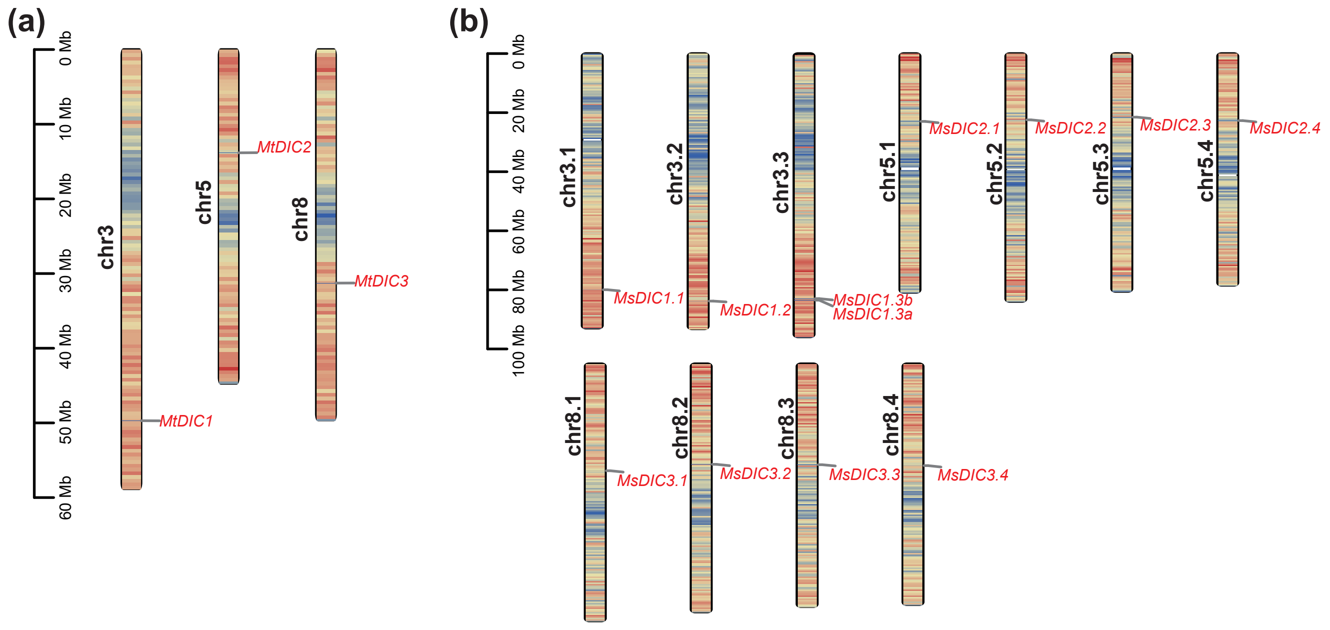

Figure S1. Chromosomal locations of *MtDIC* and *MsDIC* genes.

Figure S2

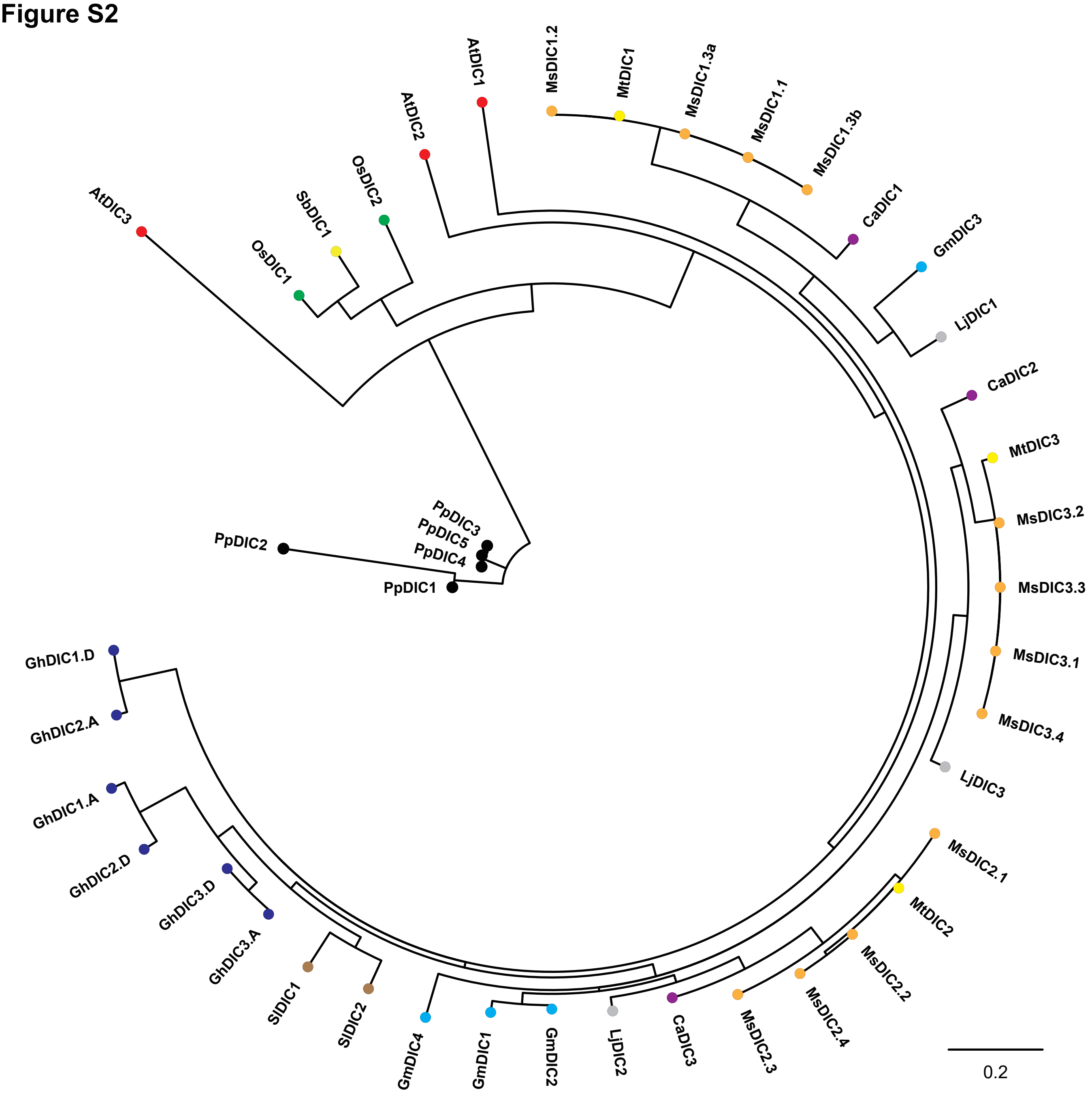

Figure S2. The maximum likelihood phylogenetic tree of the DIC gene family. DIC protein sequences from *M. truncatula*, *M. sativa*, Arabidopsis, *L. japonicus*, soybean (*G. max*), chickpea (*C. arietinum*), tomato (*S. lycopersicum*), cotton (*G. hirsutum*), sorghum (*S. bicolor*), rice (*O. sativa*), and *P. patens* represented are marked by colored circles. The maximum likelihood tree was built with 1000 bootstrap replicates.

Figure S3

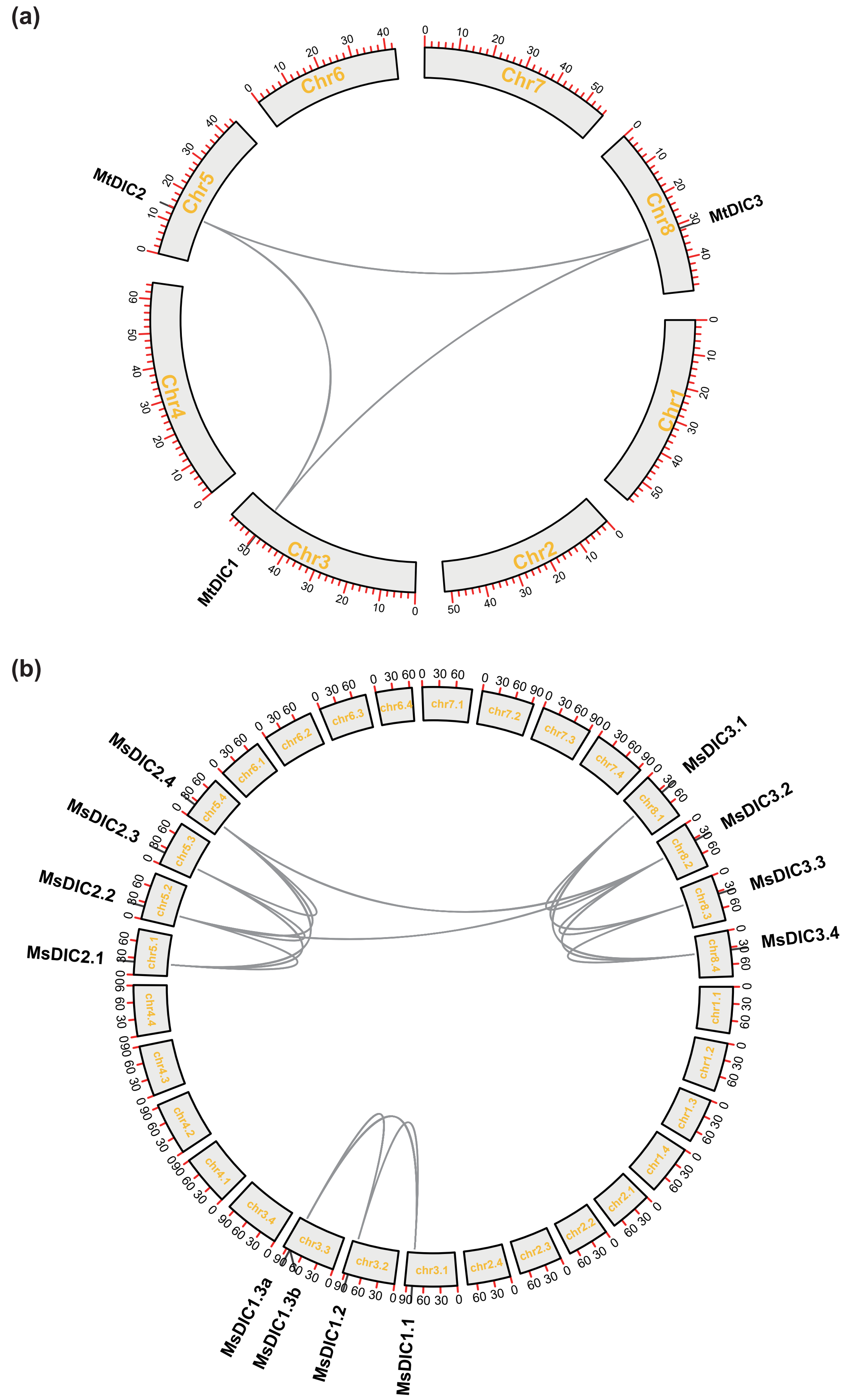

Figure S3. Syntenic analysis of the DIC gene family in the genomes of *M. truncatula* and *M. sativa*, respectively. Gray lines represent the paralog pairs.

Figure S4

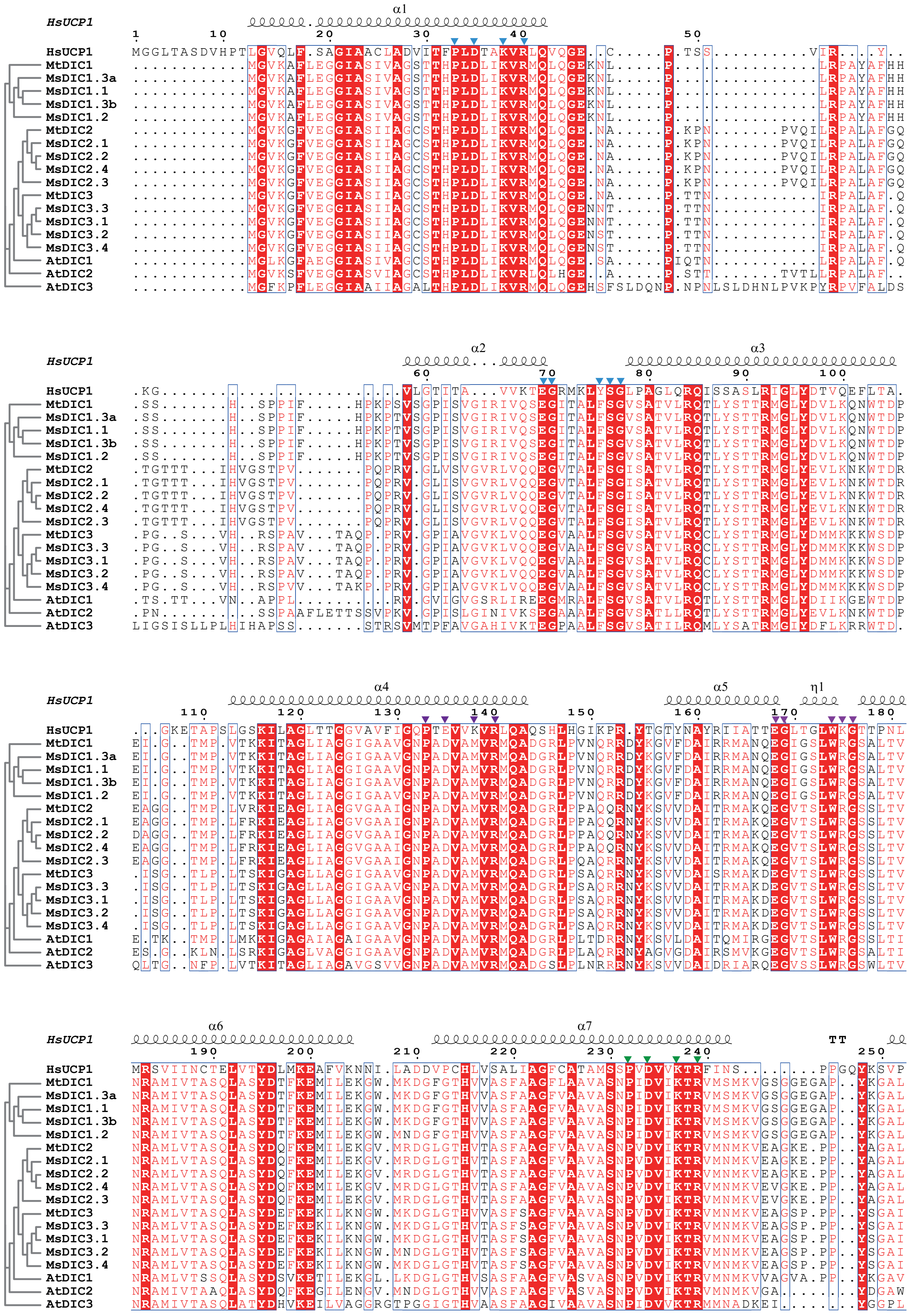

Figure S4. Multiple sequence alignment of DIC proteins. (continued on next page)

Figure S4

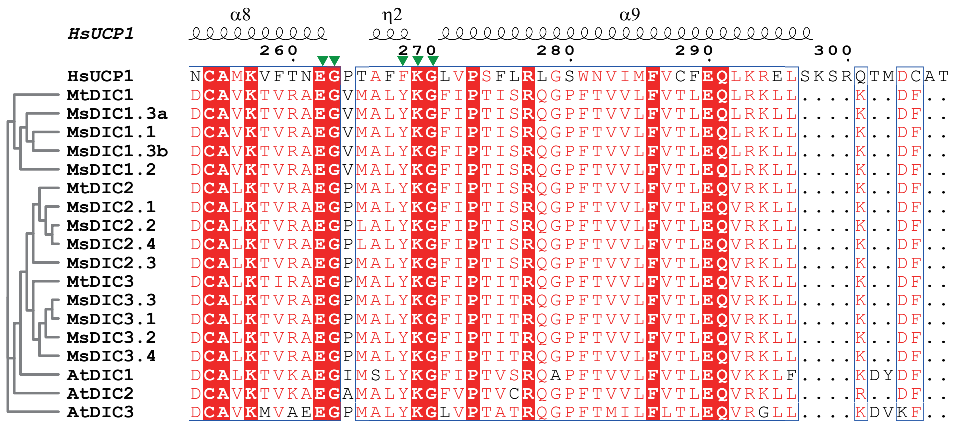

Figure S4. Multiple sequence alignment of DIC proteins. Colored triangles indicate conserved residues within the three tandem characteristic motif: PX[D/E]XX[K/R]X[K/R] (20–30 residues) [D/E]GXXXX[W/Y/F][K/R]G.

Figure S5

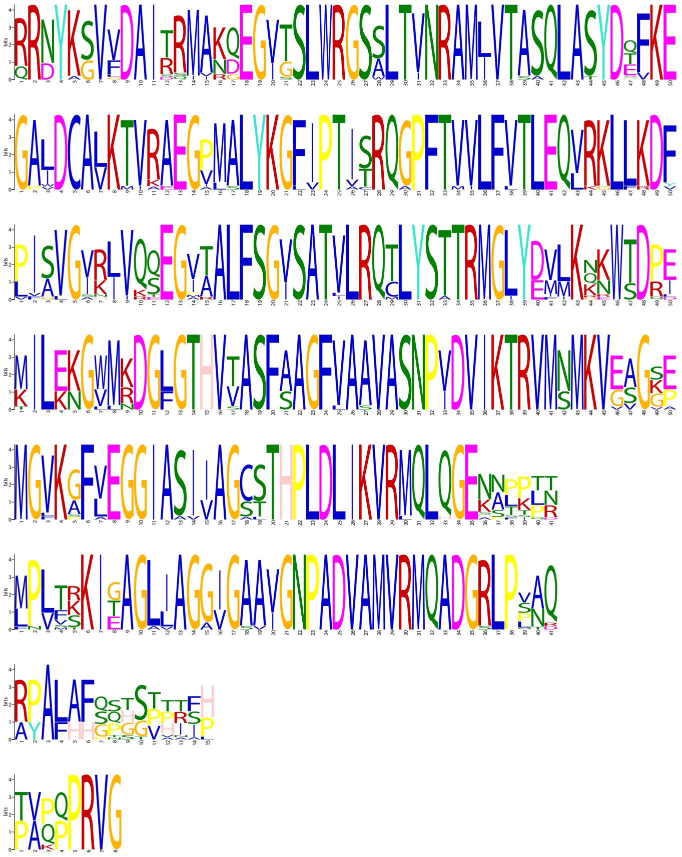

Motif	E-value	Sites	Width
1	5.2e-686	18	50
2	6.6e-686	18	50
3	1.7e-619	18	50
4	7.0e-560	16	50
5	2.2e-520	18	41
6	2.9e-473	18	41
7	1.4e-087	17	15
8	8.4e-014	10	8

Figure S5. Sequence logos of the eight conserved motifs identified in Medicago and Arabidopsis DIC proteins.

Figure S6

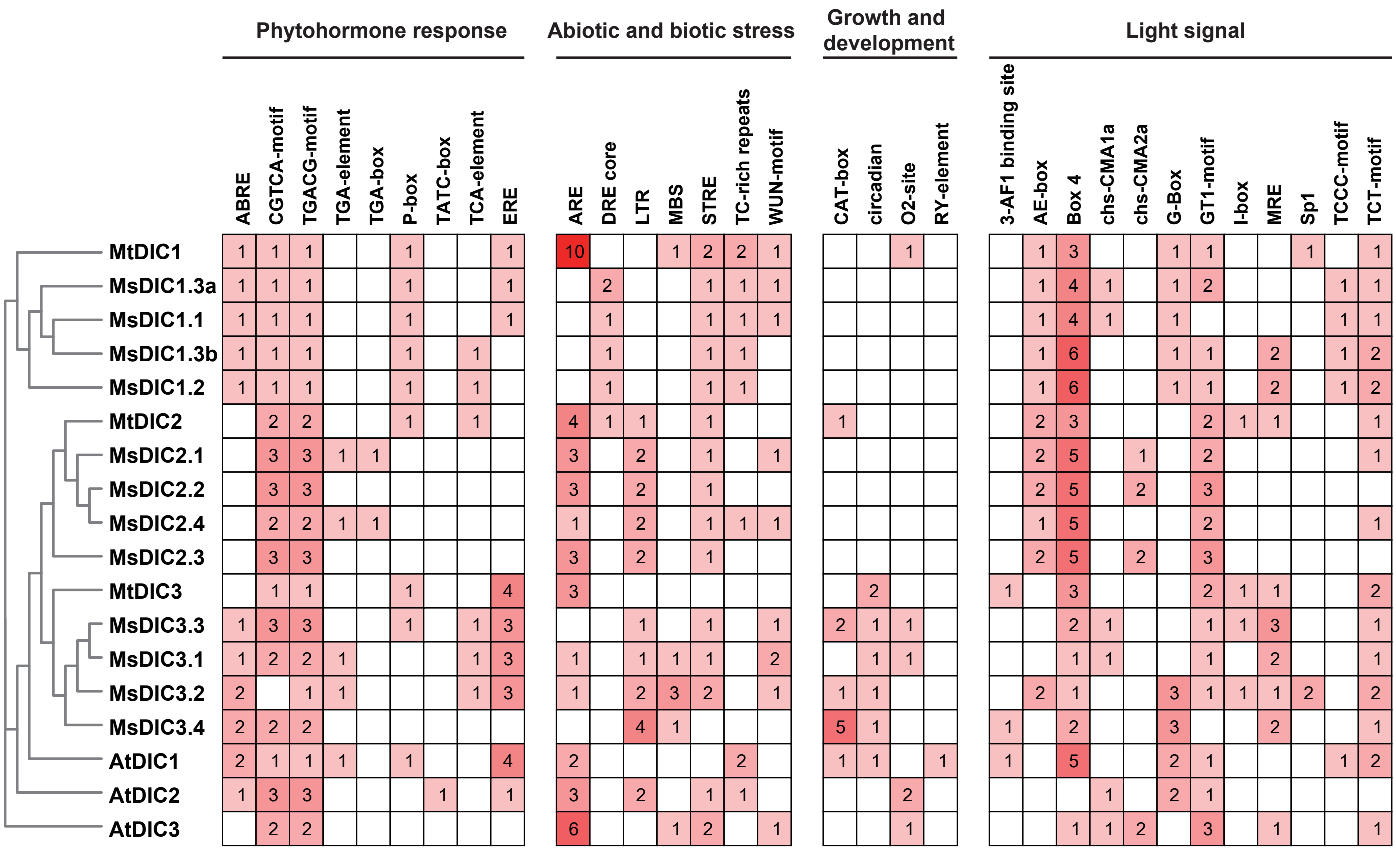

Figure S6. Heatmap showing the quantity of *cis*-acting elements in Medicago and Arabidopsis *DIC* promoters.

Figure S7

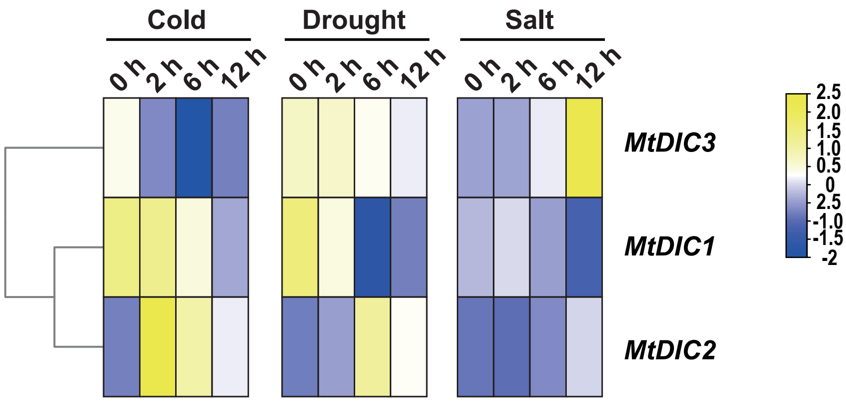

Figure S7. Heatmap of *MtDICs* expression under cold, drought, and salt stresses.

Figure S8

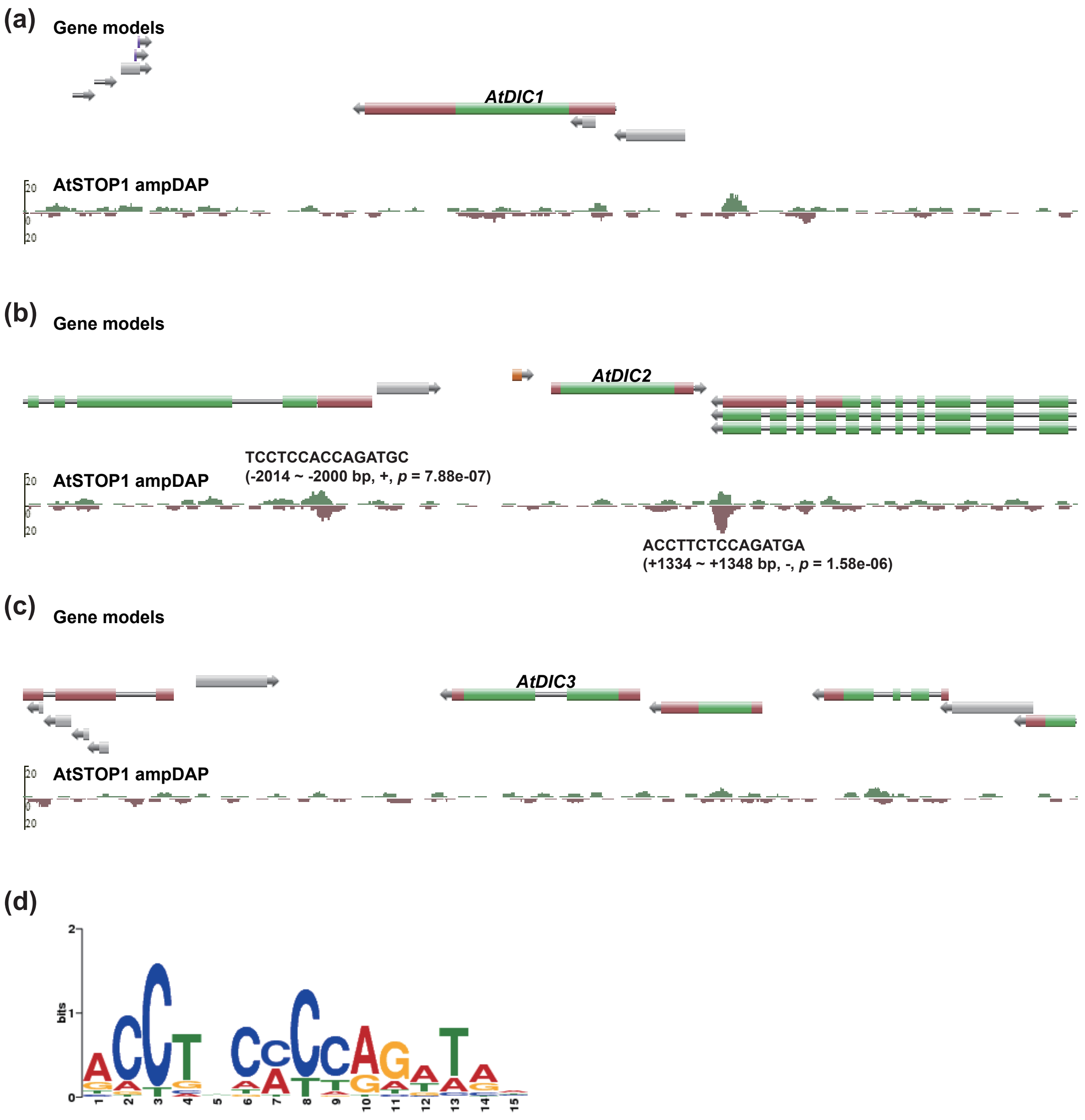

Figure S8. AtSTOP1-binding peaks detected by DAP-seq in upstream and downstream regions of *AtDIC2*. (a–c) AtSTOP1 enrichment at *AtDICs* loci. (d) Sequence logo of the AtSTOP1 binding motif.

Figure S9

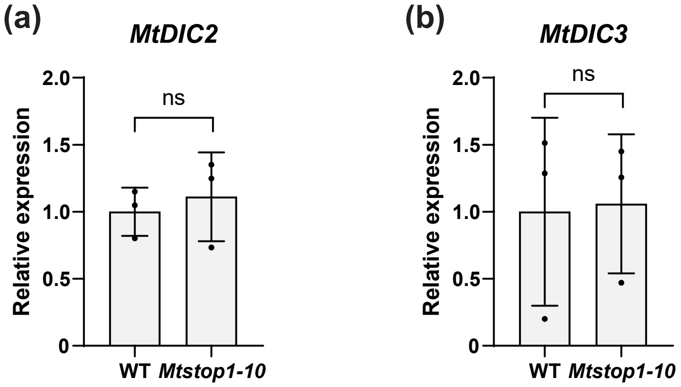

Figure S9. The expression of *MtDIC2* and *MtDIC3* in *Mtstop1* leaves remained unchanged relative to WT. qRT-PCR of *MtDIC2* (a) and *MtDIC3* (b) in leaves of WT and *Mtstop1-10* (n = 3 independent pools). Leaves from two-week-old plants; expression is relative to WT. Means ± SD; ns, not significant.

Figure S10

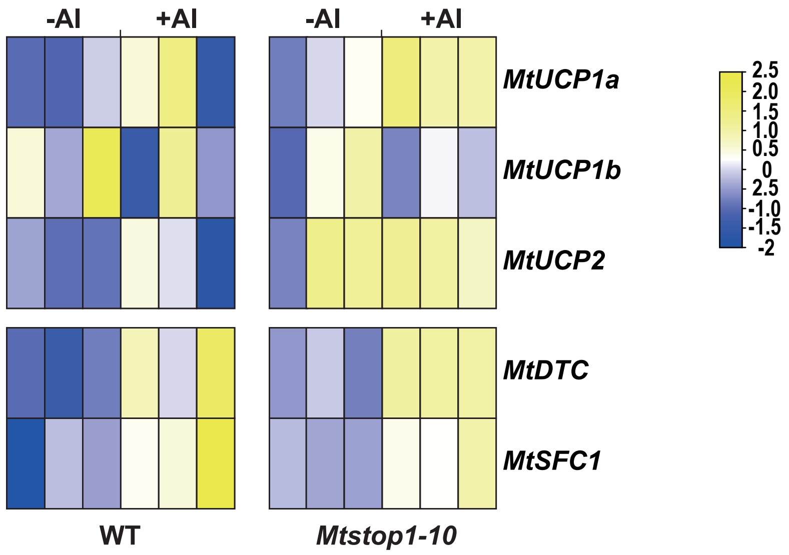

Figure S10. Heatmap of *MtUCPs*, *MtDTC*, and *MtSFC1* expression under Al stress in WT and *Mtstop1-10*.

Figure S11

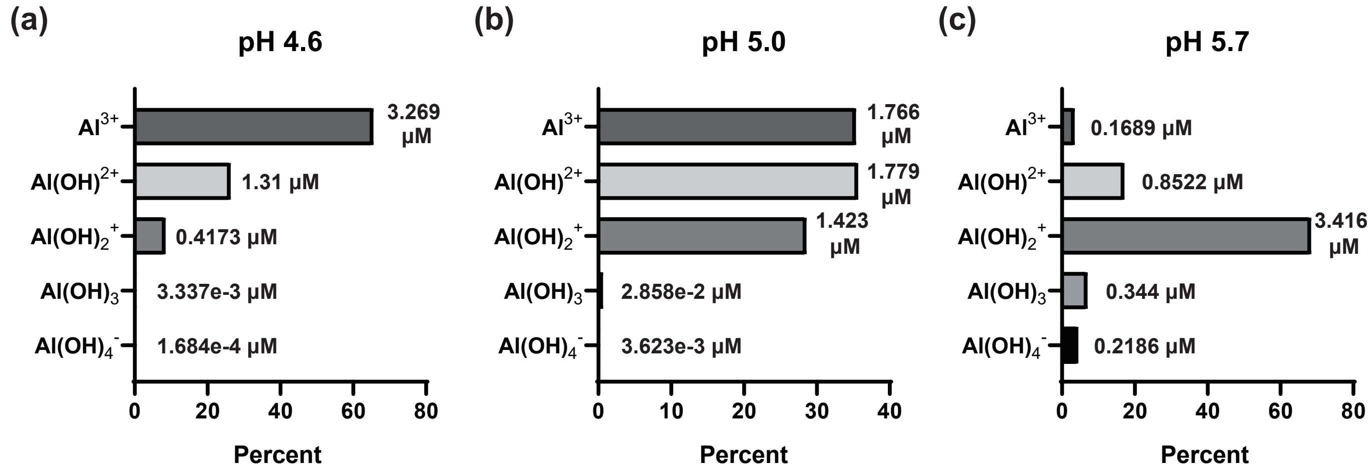

Figure S11. Effects of pH on Al speciation. The speciation of Al at various pH levels (a-c) was simulated using MINEQL+ 5.0 software. At pH 4.6, the predominant form of Al was Al^{3+}. As the pH increased to 5.7, the concentration of Al^{3+} decreased to 3.38% of the total Al content.
